# Benefits of Black Cohosh (*Cimicifuga racemosa*) for Women Health: An Up-Close and In-Depth Review

**DOI:** 10.3390/ph15030278

**Published:** 2022-02-23

**Authors:** Sradhanjali Mohapatra, Ashif Iqubal, Mohammad Javed Ansari, Bisma Jan, Sultan Zahiruddin, Mohd Aamir Mirza, Sayeed Ahmad, Zeenat Iqbal

**Affiliations:** 1 Nanotechnology Lab, School of Pharmaceutics Education and Research (SPER), Jamia Hamdard University, New Delhi 110062, India; sibanee@gmail.com; 2 Bioactive Natural Product Laboratory, School of Pharmaceutics Education and Research (SPER), Jamia Hamdard University, New Delhi 110062, India; bismakaloo123@gmail.com (B.J.); sultanpharma88@gmail.com (S.Z.); 3 Department of Pharmacology, School of Pharmaceutics Education and Research (SPER), Jamia Hamdard University, New Delhi 110062, India; asifiqubal2013@gmail.com; 4 Department of Pharmaceutics, College of Pharmacy, Prince Sattam Bin Abdulaziz University, Al-Kharj 11942, Saudi Arabia; mj.ansari@psau.edu.sa

**Keywords:** menopause, postmenopausal syndrome, women health, black cohosh, cimicifuga racemose, mechanism of action, phytomedicines, clinical trials, patents, commercial products

## Abstract

Women’s health is an imminent concern worldwide, but it remains an ignored segment of research in most developing countries, and is yet to take the center stage in even developed nations. Some exclusive female health concerns revolve around both pathological and physiological aspects. These gender-specific maladies include breast, cervical, and ovarian cancers, and physiological concerns such as menopause and osteoporosis, which are often coexistent. Recently, women’s health issues, including postmenopausal syndrome, have attracted the attention of researchers and practitioners alike, opening newer pharmaceutical research and clinical avenues. Although not counted as a disease, postmenopausal syndrome (PMS) is a female health phenomenon underpinned by hormonal depletion. Enhanced life expectancy in women has added to their suffering, and pharmacological interventions are needed. Amongst the available treatment modalities, the use of numerous botanicals has emerged as an efficient health management tool for women. *Cimicifuga racemosa* (CR or Black Cohosh) is a plant/herb which has been traditionally exploited and extensively used by women. This review is an attempt to compile and provide a summary of the importance of CR in complementary and alternative therapies for the improvement of various disorders related to women, such as menopausal syndrome, mammary cancer, and osteoporosis. It aims to systematically highlight the bioactive constituents, pharmacology, pharmacokinetics, therapeutic potentials, quality control processes, chromatographic techniques, and possible mechanisms of action of clinically effective phytomedicine for women’s health. Various clinical trials and patents relating to CR and women’s health have been collated. Furthermore, the plant and its related products have been considered from a regulatory perspective to reveal its commercial feasibility. The present review summarizes the existing data on CR focusing on women’s health, which can help to introduce this traditional phytomedicine to the world and provide some reference for future drug development.

## 1. Introduction

Menopause is a natural health concern among women all over the world, and can be better understood as a natural transition period in reproductive females. It is characterized by dramatic hormonal changes, and frequently includes social changes. It also likely changes the physical and mental needs of an adult female. Low estrogen levels as a result of ovarian dysfunction are an important characteristic feature found in postmenopausal women. This variation in hormones may lead to a diverse range of indications, collectively called postmenopausal syndrome (PMS) [1]. It is expected in all fertile women in the later stages of life, and accompanies much hardship. According to a study, there will be 1.2 billion menopausal and postmenopausal women by the year 2030, with an additional 47 million new entrants per year [2]. It has been testified that, in the developing world, maternal mortality rate is decreasing, and the life expectancy of women is increasing continuously. Therefore, attention needs to paid to women’s health in order to improve quality of life (QoL) after menopause [3]. Hormonal replacement therapy (HRT), commonly known as menopausal hormone therapy (MHT), is an extensively used treatment for postmenopausal syndrome, but its associated risks outweigh the benefits in long-term use, and it can be contraindicated in situations where women suffer from many restrictions in continuous use [4,5]. Another therapy comprises low-dose anti-depressants, serotonin, and norepinephrine reuptake inhibitors, such as Gabapentin, but none come without the burden of side effects and other pharmaco-technical limitations [6]. The detrimental effects of HRT have forced many postmenopausal women to search for a natural alternative (non-hormonal phytoconstituent) for this multifaceted problem.

Phytomedicine has been used in traditional medicine for centuries. Parts such as the flower, fruit, seed, root, rhizome, leaf, and bark are used for this purpose. Indigenous communities (such as Native Americans and Africans) used herbs in their rituals for healing. Ancient Chinese and Egyptians also used medicinal plants as early as 3000 BC. Ayurveda is a medical system primarily practiced in India that has been known for using botanicals for nearly 5000 years. Unani medicine is another known system of traditional medicine that has helped mankind for centuries by alleviating diseases using drugs derived from plant origin. Nowadays, with herbalism becoming more mainstream, there is continuous upgrading/improvements in analysis and quality control along with advances in clinical research, which add value to the botanicals in treating and preventing disease. The categories are drugs (e.g., Ayurveda and Unani), complementary and alternative medicines (CAM), dietary supplements, and novel foods. There is a range of botanicals that are extensively used for several health problems, with either curative or supportive potential, that add a quantity of referenced data related to global consumption and trade. One such botanical is *Cimicifuga racemosa*/*Actea racemosa* (CR) which is extensively used to treat women’s health-related issues, and also carries a lot of traditional uses [7]. CR has been used for over 40 years in Europe for the treatment of menstrual pain.

CR is native to eastern and central North America, and corresponds to the family Ranunculaceae. It is also distributed in Canada and China, and cultivated in Europe. It is often called black cohosh, bugbane, rattle weed, snakeroot, squaw root, or rheumatism weed [8]. Furthermore, various synonyms of CR, according to the International Plant Name Index, are listed as *Actaea racemosa* L., *Actaea monogyna* Walter, *Actaea gyrostachya* Wender, *Botrophis pumila* Raf, *Botrophis serpentaria* Raf, *Botrophis actaeoides* Raf. ex Fisch. and C.A. Mey, *Cimicifuga americana* Muhl, *Cimicifuga serpentaria* Raf, and *Thalictrodes racemosa* (L.) Kuntze [9]. The root and rhizome of CR have traditionally been used in the management of ailments such as rheumatism, malaria, sore throats, colds, and complications associated with childbirth [10]. It has been used for centuries by Europeans for treating menopausal symptoms, and a recent clinical study also supports the safety and efficacy of CR [11,12]. In contemporary Western herbal medicine, the use of CR is usually restricted to the management of menopausal symptoms and other difficulties related to the reproductive system in women [13]. Currently, CR is widely used to mitigate menopausal symptoms such as hot flashes, night sweats, sleep disturbances, vertigo, nervousness, mood swings, and vaginal dryness associated with postmenopausal females [14]. CR was listed among the ten bestselling OTC (over-the-counter) herbal remedies in America in the year 2018 [15,16,17]. Currently, CR-containing preparations are marketed in huge quantities in the United States and Europe under various brand names, such as Remifemin, Cimicifuga-Oligoplex and Cimicifuga Pentakran. Some of the products available on the US market have been listed in Table 1 [18].

This herbaceous perennial plant has a long history of treating varieties of ailments with global demand. Nowadays, it is an internationally admired herbal remedy for the treatment of menopausal symptoms. In Germany, CR extract has been marketed since 1956, and the technical data on this herbal product has been included in several monographs, including American herbal Pharmacopoeia 2002, British Herbal Compendium 1992, British Herbal Pharmacopoeia 1996, British Pharmaceutical Codex 1934, Complete German Commission E (equivalent to US FDA), Martindale 32nd edition, Mills and bone, WHO volume 1 (1999), and PDR of herbal medicines 2nd edition [19,20]. According to the European medicines agency (EMEA) and the committee of the herbal medicinal product (CHMP), the rhizome of CR is a well-known herbal alternative for human consumption and has a therapeutic indication for alleviating minor neuro vegetative complaints associated with menopause, such as hot flashes, sweating, etc. [21]. In Canada, CR rhizome is legally recognized as an active ingredient of registered natural health products intended for oral use [22]. Therapeutic uses include pain associated with menstruation, relief of premenstrual symptoms, and relief of symptoms associated with menopause and muscle and joint pain associated with the rheumatoid condition. It is included in the category of herbal remedies or dietary supplements [23]. Many clinical, preclinical, in silico and in vitro studies confirm that the aqueous or ethanolic CR extract does not contain an estrogenic compound, rather it has neurotransmitter-like activities which positively influence postmenopausal syndrome.

In this review, an attempt has been made to accentuate and summarize the significance of CR in complementary and alternative treatments for the improvement of postmenopausal syndrome, together with its capability as a natural resource as an alternative to HRT. Furthermore, we aimed to systematically highlight CR’s bioactive constituents, pharmacology, pharmacokinetics, therapeutic potentials, quality control processes, chromatographic techniques, and possible mechanisms of action towards clinically effective phytomedicine for women’s health. This review also highlights various clinical trials and patents relating to CR and women’s health. Furthermore, it tries to compile the various health concerns associated with CR with the perspectives of different regulatory agencies, and list some of commercially available products of CR. The present review is also an attempt to gather existing data on CR with a main focus on women’s health, specifically menopause and related problems, which might help in introducing this traditional phytomedicine to the world, and provide some reference for future drug development.

## 2. Phytochemicals Constituent of CR

Phytochemicals, otherwise known as secondary metabolites, have significant biological activity and are also responsible for the color and odor of plants. Many secondary metabolites such as alkaloids, flavonoids, sterols, triterpenes, etc., play major roles in nutrition, physiology, and the management of various ailments [24,25]. They represent some of the most distinctive classes of compounds in higher plants, and may be classified based on chemical structure, composition, or solubility in various solvents or pathways by which they are synthesized.

Components of plants explored for phytochemical constituents comprise of the underground (roots and rhizomes) as well as aerial parts (stems, leaves, flowers and fruits). Phytochemicals in CR rhizomes have been well studied, and the key constituents are triterpene glycosides, phenols, flavonoids and alkaloids, etc. CR also contains aromatic acid (ferulic acid, iso ferulic acid, caffeic acid and caffeic acid methyl esters), cinnamic acid esters (cimicifugic acid, cimicifugic acid A–F, cimiracemates A–D, fukiic acid, piscidic acid and fukinolic acid), resin, phytosterol, fatty acid, starch, and sugar as minor compounds [26,27]. It contains some alkaloids such as quinoline and quinolizidine types, anagyrine, baptifoline, magnoflorine, methyl cystine, methyl serotonin, etc. Some of the alkaloids are also undefined, which are present in minor quantities. Other constituents include citrullol, gum, resin, tannins, phosphoric acid, starch, phytosterol, cholines, and Betaine.

### 2.1. Triterpenoids

Triterpene glycosides are a large, structurally distinct group of chemicals obtained from the metabolites of isopentenyl pyrophosphate oligomers, and signify the largest group of phytochemicals. Triterpene glycoside conjugates accrue in plants and produce saponins. Triterpenes and saponins have been shown to possess a range of biological actions such as anti-inflammatory and anti-cancer effects, and can promote or induce apoptosis [24,28]. These are the main class of compounds found in CR extract. CR triterpenes have a five-ring structure, the same as that of the four-ring structure of steroids. The majority of triterpene glycosides have 9, 19-cycloartane triterpene skeletons with different substitutions. The position and varieties of chemical substitution are responsible for different stereochemical configurations. Moreover, 23-epi-26-deoxyactein (previously known as 27-deoxyactein), actein, and cimiracemoside A are commercially available triterpenes, and are commonly used for the standardized CR extract.

As with many botanicals, CR is complicated to study due to the absence of standardization of the extract to one or more active ingredients. Certainly, in CR, the active components are ambiguous; nonetheless, the triterpenes of CR are thought to be responsible for its biological action, and hence are used for the relief of PMS [29,30].

Furthermore, the majority of investigations on efficacy have been carried out on whole extracts or standardized extracts of CR with respect to triterpenes. Although many components are found in the extract, which components are actually necessary for the symptomatic relief of menopause is still unknown.

Triterpenes are one of the most important constituents present in hydroalcoholic extracts of CR. Primarily, the bioactive constituents of CR extract are supposed to be found in the triterpene glycoside fraction. So far, more than 40 types of triterpenes have been isolated from CR extract [31,32]. To distinguish triterpenes from each other is a major challenge because of their complexity and structural similarities. Actein and 23-epi-26-deoxyactein are the highly abundant triterpenes found in the root and rhizome of the CR, and are frequently used as standardization markers for CR formulations [33,34]. Quantization of the triterpene glycosides is generally difficult because they do not have a chromophore that absorbs light above 200 nm, thus limiting their sensitivity and the ability for UV analysis. Thus, HPLC-evaporative light scattering detection (HPLC-ELSD) has become the most accepted technique used for the quantitation of the triterpene glycosides in CR [34], but ELSD has some limitations such as poor sensitivity, highly non-linear calibration curves, and poor reproducibility. Table 2 enumerates various chromatographic techniques for the evaluation of CR triterpenes.

### 2.2. Phenolic Constituents

The main phenolic components of CR are caffeic acid, hydroxycinnamic acids, ferulic acid, and isoferulic acid. Other phenolic constituents separated from the extract of the CR roots and rhizomes include rotocatechuic acid, fukinolic acid, protocatechualdehyde, methyl caffeate, p-coumaric acid, ferulate-1-methyl ester, 1-isoferuloyl-β-d-glucopyranoside, and cimicifugic acids A, B, and D–F [49]. It has been found that both triterpenes and phenols are stable for many years under controlled environmental conditions, and do not undergo considerable changes [47,50]. Furthermore, it has been found that triterpenes can maintain their stability at a variety of temperatures and humidity conditions; polyphenols, on the other hand, are only stable at room temperature and low humidity conditions. The characteristics of CR not only enhance its utility in industry, but are also very useful in clinical research.

### 2.3. Flavonoids

Many findings have found that flavonoids, such as the isoflavone formononetin, can be isolated from CR [14,33,51,52]. The presence of formononetin in the CR extract was first discovered by Jarry et al. [53] following the analysis of the extract by various analytical methods. It was believed that the pharmacological action of the plant was due to the presence of formononetine which helps to alleviate menopausal distress in adult women by its estrogenic activity. The presence of formononetine from the methanolic extract of rhizomes and roots of CR was confirmed by using TLC fluorometry [54]. The research, however, was unable to identify the compound in raw plant materials or extracts. Struck et al. [51] were unable to identify the compound in the ethanolic and isopropanolic extract of the CR. Li et al. also failed to identify formononetine from the methanolic extract by using an HPLC–photodiode array detector (HPLC-PDA) and an HPLC-ELSD [27]. Later on, Kennelly et al. and Jiang et al. [45,55] also failed in identifying the presence of formononetine in the commercial CR products and rhizomes by using various analytical techniques in separate experiments.

### 2.4. Alkaloid

Alkaloids are important constituents of natural products that have established biological action, principally as CNS agents. There are more than a hundred nitrogenous compounds in CR extract [56]. It contains isoquinoline, indole, and guanidine-type alkaloids. The presence of guanidine-type alkaloids in the extract is one of the distinctive characteristics of the CR metabolome. Phenolic acids present in the extract may possibly behave as counter ions to positively charged alkaloids and create strong ion pairs that are responsible for the biological activity of the extract. Nω-methylserotonin (indole alkylamine) is a compound that is found in CR extract, and it has the active serotonergic principle [57,58]. It binds to the serotonin transporter, and hence may be considered as a selective serotonin reuptake inhibitor, which may contribute to the biological activity of CR extract in menopause. The preceding fact suggests that alkaloids are most likely the active components accountable for the perceived CNS action of CR.

Most of the research work focuses only on triterpenes as the active ingredient in CR extract, which are believe to be responsible for pharmacological action. Table 3 describes the pharmacologically active constituents of CR from the roots and rhizome of the plant. However, other types of CR ingredients may be explored for their pharmacological activity. It is also important to investigate the other part of the plant apart from the root and rhizome to find out the most active ingredients.

## 3. Structure of Biologically Active Compounds

The metabolite pattern of CR is unusually complex, and is isolated from the root/rhizome/aerial parts of plant which are responsible for showing a variety of activities [31]. A few major compounds present in CR are enlisted in the Table 4.

## 4. Quality Control

CR is one of the most clinically comprehensively investigated plants, as it is widely used by elderly women for the symptomatic relief of postmenopausal symptoms, an alternative to estrogen therapy [18]. Due to a large variety of complex phytochemical entities, species identification remains very taxing. There are a number of cimicifuga species, having very similar or even identical constituents as that of CR, that have been used in place of it [64]. However, it has always been imperative to identify the correct species and segregate the adulterate one. This is particularly important when the extract is used to investigate clinical actions in the laboratory for in vitro, in vivo or clinical trials. This is because a proper and authentic extract will help to produce reproducibility of the result, facilitating the accomplishment of all regulatory requirements. The identification of the extract of various cimicifuga species may be performed by using traditional pharmacognostic techniques such as taxonomical and organoleptic identification. Other methods of identification of the species include RAPD-PCR analysis [66], DNA fingerprinting [67], and FT-IR spectroscopy. Although the FT-IR spectroscopy technique appears to be a popular technique in the industry, it can be significantly affected by the presence of the excipients milieu. However, chromatographic methods remain the most dominant technique used for the identification of the component, as this method can provide both qualitative and quantitative analyses, and is able to provide detailed chemical information by combining with the UV/ELSD detector and/or MS spectrometry. Table 5 enlists various quality control methods available for CR, which helps to distinguish this particular species from others.

## 5. Pharmacology and Biochemistry of CR Extract/Biological Characterization of CR

### 5.1. Management of Menopausal Syndrome

Due to the severe side effects associated with HRT, many women have turned to alternative therapies such as the use of complementary and alternative medicines for the alleviation of menopausal symptoms. Generally speaking, there is a recent predilection towards herbal remedies which encourages the exploration of this domain to find newer moieties for the management of menopause [74]. For some years, various investigations have been carried out to ascertain the effect of CR on menopausal symptoms. The pharmacology of the rhizome extracts of CR has been investigated thoroughly but, irrespective of extensive clinical trials, the active ingredient and mechanism of action of CR is still ambiguous. Various studies have yielded conflicting results regarding the effect of CR on human physiology, which are summarized as follows.

It has been suggested that CR possesses direct estrogenic activity; it is widely used by menopausal women considering its estrogenic activity, as it possesses functions similar to estrogen, such as the alleviation of hot flashes, the reduction of depression, and the possibility of providing protection against bone loss [75,76]. The literature suggests there is a compound present in CR extract, identified as formononetin (isoflavones phytoestrogen), which is responsible for the estrogenic activity [53], but later reports failed to recover this compound in CR [51,55], and hence declined the role of its estrogenic activity [77]. Research has shown that the CR extract possesses tissue-specific action that behaves as an estrogen agonist in certain tissues [12,78,79] and as an estrogen antagonist in other tissues [80,81]. As a result, it is possible to conclude that CR extract acts as a selective estrogen receptor modulator (SERM). An ideal SERM can be defined as one that behaves as an estrogen on bone and brain but does not act as same in the uterus and breast. Hence, compounds in CR extract may persuade the SERM criteria.

However, recent studies support the limbic action that is the action on the hypothalamus. More specifically, this extract contains substances with neurotransmitter-like activities which are beneficial for the symptomatic relief of postmenopausal syndrome [82]. It also has a central activity rather than a hormonal effect [83,84,85]. Triterpene glycoside, which is a major constituent of CR, does not bind with the estrogen receptors and thus does not exert an estrogenic effect [7]. It functions in a serotonergic manner rather than an estrogenic manner, and might act on 5-HT_1A_, 5-HT_1D_, and 5-HT_7_ receptors [58]. Data support the presence of a compound called Nω-methyl serotonin in CR extract which is responsible for its action on serotonergic receptors by acting on 5-HT_1A_ and 5-HT_7_ receptors [57,86]. It may possess selective serotonin reuptake inhibitors (SSRIs) activity. SSRIs are efficient in alleviating hot flashes in menopausal women, but have some side effects. CR extract binds with the serotonin receptor, most intensely on serotonin receptor 5-HT_7_ and 5-HT _1A_ as a mixed competitive ligand. Both these receptor subtypes are involved in thermoregulation in the hypothalamus [58,87]. 5-HT_1A_ interacts with the serotonin transporter in the hypothalamus to regulate serotonin re-uptake. So, CR extract may contain substances which by this mechanism alleviate postmenopausal hot flashes. Another research claimed the action of CR extract is due to its affinity to the human µ opiate receptor ([3H] DAMGO) [88].

CR extract can be successfully used to treat dry mouth which increases appreciably after menopause. The efficacy of CR in treating dry mouth was compared with that of estrogen in an ovariectomized rat model. It was found that both estradiol and CR had a protective effect on the animal’s sublingual gland, but the exact location and mechanisms of action that produces these actions are different [89]. Furthermore, the possible mechanisms by which the estrogen and standardized isopropanolic CR act on the submandibular gland in postmenopausal animal models were investigated [90], and finally, it was concluded that both can alleviate menopausal oral dryness. However, they possess different mechanisms of action.

Although accurate mechanisms underlying the actions of CR have not been determined, its medical effects are primarily related to triterpene glycosides [26,91,92,93,94], and also may be due to the multiple synergistic effects of unknown constituents present in it. Table 6 and Table 7 list various clinical trials and patents of CR relating to women’s health.

**Figure 1:**
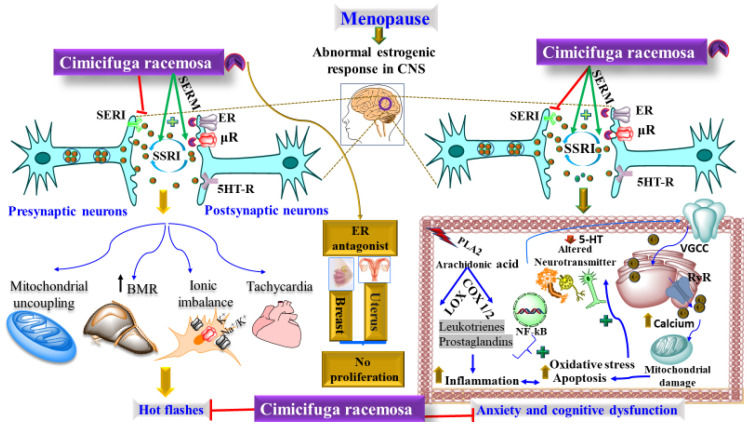
Showing the proposed mechanism of action of CR in menopause-related hot flashes, anxiety, and cognitive dysfunction.

The discussion in the literature crystallizes into a collective action of CR where different pathways can be elucidated. Figure 1 showcases a collective effect of CR through various pathways. It could be henceforth deduced that CR may follow a similar bio fate like other moieties, and may lead to the arrest of symptoms such as hot flashes, anxiety, and cognitive dysfunction. It is further anticipated that alteration in the level of serotonin during postmenopause, either due to the altered activity of serotonin transporter (SERT) or increased serotonin reuptake, diminishes the level of serotonin considerably at the postsynaptic neuronic 5HT-receptor (5HT-R). This drastic shift in the level of serotonin along with an alteration in the level of circulating estrogen results in mitochondrial dysfunction, increases basal metabolic rate (BMR), and causes ionic imbalance, tachycardia, and thermo-dysregulation, leading to hot flashes. Furthermore, the reduced level of serotonin as well as estrogen also causes cognitive dysfunction. During the normal physiological condition, a sufficient level of serotonin and estrogen maintains the normal physiology of CNS and impedes neuronal stress, lipid peroxidation, and neuroinflammation. However, during postmenopause, the absence of these two neurohormones triggers the rate of lipid peroxidation in neuronal polyunsaturated fatty acids (PUFA) via oxidation of arachidonic acid (AA), damage to neuronal architecture, and dysregulated calcium level via voltage-gated calcium channel (VGCC), disturbed endoplasmic reticulum and mitochondrial dysfunction. These attributes cumulatively cause neuronal oxidative stress, neuroinflammation and neuronal apoptosis leading to anxiety and cognitive dysfunction. However, CR by virtue of its polyvalent mechanism of action such as antioxidant, anti-inflammatory, anti-apoptotic, as a potent SSRI, agonist to μR and estrogenic receptor (ER) acts as a pivotal player. Additionally, CR also acts as an antagonist to ER on the uterus and breast and has shown no evidence of cellular proliferation; hence, CR could be a potential therapeutic alternative against post-menopausal-related hot flashes, anxiety, and cognitive dysfunction. This, seemingly, is an advantage of CR over other emulates.

The role of 5-HT in the postmenopausal-induced hot flashes and anxiety can be validated from the studies where the administration of selective serotonin reuptake inhibitor (SSRI) revealed a considerable reduction in the frequency of hot flashes and anxiety. Paroxetine, fluoxetine (SSRI), and venlafaxine (serotonin, norepinephrine reuptake inhibitor) have been reported to mitigate the frequency of hot flashes considerably.

### 5.2. Management of Postmenopausal Osteoporosis

Osteoporosis is usually documented as a significant public health concern, specifically amongst postmenopausal women. This disorder is characterized by compromised bone strength resulting in an enhanced possibility of fracture. Due to osteoporosis, the bone mineral density decreases, which leads to the structural depreciation of tissue and results in fragile bones. It is one of the most prevalent diseases in menopausal women, and is strongly linked with poor QoL. It has also been reported that the isopropanolic extract of CR (remifemin) may facilitate postmenopausal osteoporosis [111,112]. It protected bone structure by preventing the loss of bone density and reducing bone reabsorption in an ovariectomized rat model [112]. Additionally, it showed a selective estrogen receptor modulator (SERM) mechanism by exerting estrogenic properties in the bone tissue (mainly in osteoblasts) and fat tissue, but not in the uterus, of an ovariectomized rat model [12,79]. One of the studies claimed that the triterpenoids of CR inhibited osteoclastic bone resorption by suppressing both the formation of osteoclast-like cells and their resorbing activity, thus increasing the bone mineral density in an ovariectomized mice model [76,113]. Another study alleged that triterpene-saponin-fraction slowed the progression of osteoporosis, most likely by lowering the fat load of bone marrow and probably by lowering pro-inflammatory cytokine secretion [111]. This investigation claimed that actein and deoxyactein, which are the major triterpenes found in CR extract, had a positive effect on the skeletal structure of postmenopausal women. Actein protects the bone by preventing oxidative damage to osteoblasts in osteoporotic patients and deoxyactein results in a considerable rise in the growth of cells, collagen content, alkaline phosphatase action, and mineralization in the cells, thus protecting bone density [114,115,116,117].

**Figure 2:**
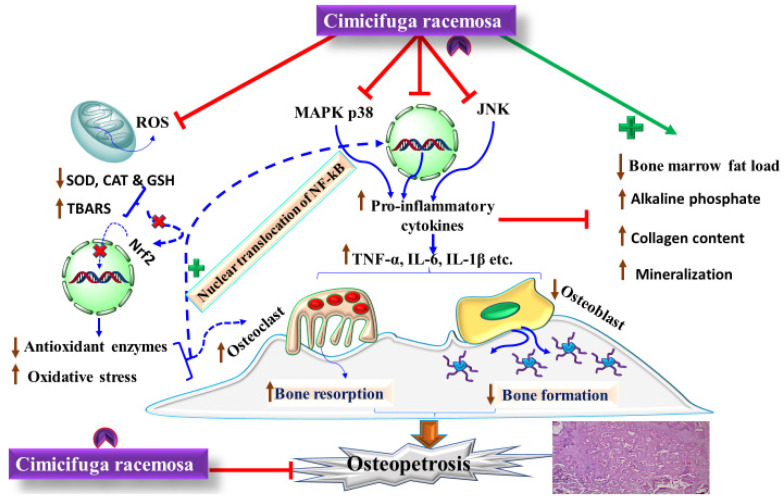
Showing the proposed mechanism of action of CR in post-menopausal-induced osteoporosis.

Figure 2 shows the proposed mechanism of action of CR in post-menopausal-induced osteoporosis. Osteoporosis is complex and multifactorial in origin. Several confounding factors work together and initiate as well as progress the cascade of osteoporosis among postmenopausal women. During the normal physiological condition, the optimum level of circulating estrogen maintains the equilibrium between osteoclastic and osteoblastic activity, and hence maintains the structural integrity of the bone. However, when the level of circulating estrogen reduces significantly, as seen among postmenopausal women, increased oxidative stress with improved reactive oxygen species (ROS) production, high thiobarbituric acid reactive substances, and the reduced activity of superoxide dismutase, glutathione, and catalase have been observed. Moreover, a reduced level of estrogen triggers the activities of inflammatory transcription factors such as mitogen-activated protein kinase p38 and c-Jun N-terminal kinase. Later, these are translocated into the nucleus and regulate the production of proinflammatory cytokines such as IL-6, IL-1β, TNF-α, etc. Thus, enhanced oxidative stress and inflammatory cytokines regulate the osteoclastic activities and downregulate osteoblastic activities leading to osteoporosis. CR, by virtue of its anti-oxidant, anti-inflammatory and estrogenic-like action, effectively ameliorates osteoporosis, and hence can be a potential therapeutic alternative for postmenopausal-mediated osteoporosis.

### 5.3. Adjuvant Treatment in Mammary Cancer

It has been found that extracts of CR enriched with triterpene glycosides may have chemopreventive potential, and can be successfully used in mammary cancer patients without having adverse effects on breast tissue [118]. Studies have also shown that there has been neither an increase in mammographic breast density, nor any enhancement in breast cell proliferation in naturally postmenopausal women with climacteric complaints [98,101]. However, one of the systematic reviews concluded that there was no association between CR and a reduction in hot flashes in mammary cancer patients owing to the lack of sufficient evidence, but demanded further research [119]. Tamoxifen frequently induces or worsens menopausal symptoms in breast cancer patients receiving antioestrogen therapy. As estrogen replacement is contraindicated, herbal alternatives such as extracts of CR are frequently used. It has been proven that CR extract can be tolerated in mammary cancer patients receiving tamoxifen therapy, showing predominant psychovegetative indications [17,102,120,121,122,123,124].

One of the latest studies claimed that CR revealed significant anti-cancer properties on the expression of PR, ER-α, and BRCA1 in MCF-7 and T-47D mammary cancer cell lines. Furthermore, they revealed that in presence of CR, the proliferative action of estrogen was decreased, hence altering the growth of hormone-dependent mammary cancer cells [125]. Another study investigating the effects of standardized CR extract and its key triterpene actein on growth rates and the metabolism of the steroid hormone in human breast cancer cell lines concluded that it did not promote cell growth in breast cancer cell lines, or have any impact on estrogen concentration. Alternatively, they stimulated androgen formation, which might contribute to improved menopausal symptoms in adult women [126].

### 5.4. Management of Other Diseases

In the past, CR extract has been used to deal with pain and inflammation. In Korean folk medicine, it has been widely used for this purpose. Research has been conducted on the potential action of CR extract on the allergic response in mast cells shown to inhibit the passive cutaneous anaphylaxis reaction induced by anti-IgE- in a dose-dependent manner, and also to inhibit the mRNA of cytokines (induced by inflammatory agents); It may claim the anti-inflammatory and anti-allergic effects but the anti-oxidant property of CR extract shows conflicting results [29,127,128]. Furthermore, CR extract can also be used for the treatment of several other diseases such as diabetes, neoplasia, sarcopenia and myocardial insufficiency, obesity, etc. [129,130,131,132].

## 6. Pharmacokinetics

It is a regular practice to chemically standardize the dietary supplement CR with respect to triterpene glycosides. The most abundant triterpene used for this purpose is 27-epi-26 deoxyactein [27,40,42]. Generally, it is regarded as the commercially available analytical marker for CR triterpenes. By characterizing the marker compound, a brief idea about the entire group of chemicals can be obtained. Hence, it is essential to characterize the available marker, which is always helpful. In one of the studies in the literature, the pharmacokinetics of 23-epi-26 deoxyactein following the oral consumption of standardized CR extract was extensively investigated, and the half-life of the compound was found to be approximately 2 h [133]. The compound did not undergo metabolism and was excreted as such in the urine. However, the amount excreted in the urine was low, which suggested that renal clearance was not the only primary route of clearance. The compound may have been excreted as such through bile, and degraded in the gastrointestinal tract [133]. In another study, it was found that the triterpene glycosides of CR exhibited pH-dependent solubility with the highest concentration at pH 7.5. It possessed a rapid dissolution profile, high permeability through Caco-2 monolayers, and good absorption capacity through the duodenum, jejunum, ileum and colon. Triterpene possesses high permeability, and can be categorized as BCS class I (high solubility, high permeability) [134].

## 7. Health Risks

There have been many clinical trials conducted to treat menopausal symptoms using various CR preparations, showing that the herb CR, which is commonly used to mitigate climacteric complications, is associated with a low occurrence of adverse effects [135,136,137,138]. Although it does not show causality in all cases, the most commonly reported side effects are related to the liver. There was also a lack of analysis regarding the authentication of CR in the commercial products employed in this study. This problem raises the question of impurities and adulterants in some CR products used. Table 8 list the viewpoints of different regulatory agencies/professional bodies regarding liver toxicity relating to CR.

So, it may be concluded that the possible instances of hepatotoxicity may be caused by adulterants, impurities, or wrong Acteae species in the employed CR products. Moreover, the use of CR may not pose an explicit liver toxicity threat, but quality challenges in some products may be responsible for this predicament [17]. Accordingly, these products should be independently analyzed to confirm the existence of these problems. Additionally, these problems can be circumvented by the intervention of additional regulatory quality specifications. Furthermore, the majority of the investigations have analyzed CR use for short intervals, usually 6 months or less, so the long-term safety of CR in humans is still questionable and demands long-term reliable clinical trial outcomes.

## 8. Future Prospective

Although the majority of the chemical constituents of CR root are known, it possibly contains many more which remain to be unraveled. There is very scant data on the chemical constituents of the aerial parts of CR, and the exploration of this area would add great scientific value. Furthermore, it is pertinent to adopt reliable research tools to universally ascertain the effective dose of CR triterpene. This would help in extrapolating its use in clinical settings. Although many RCTs have revealed the effectiveness and safety of CR for the amelioration of PMS, the exact mechanism of action is yet to be established. Based on the pharmacological data and understanding from the availability of the associated literature reports, the authors have attempted to trace the MoA of CR for a better understanding. It may also be submitted that the CR could well have a larger umbrella of therapeutic actions, and has a possibility of being indicated thereof. Another pharmaceutically relevant dimension is to explore its efficacy via alternative routes, such as transdermal or intranasal, in addition to the oral administration which has already been reported. The blood–brain barrier activity of the extract, if ascertained, would additionally help pharmaceutical scientists in designing appropriate NDDS.

Moreover, enough research to assess live toxicity post oral administration needs to be collated. CR has emerged as a popular and potent plant for the management of climacteric changes in adult women, and hence it is advisable to carry out extensive studies in order to garner appreciable support for its prolific clinical use across the length and breadth of the female population across the globe.

## 9. Conclusions

A thorough literature profiling suggests that CR is more efficient compared to a placebo in treating vasomotor symptoms resulting from natural menopause; however, it is not significantly better than an oral estrogen and progesterone combination (O+P) [139]. Transdermal O+P is the most successful therapy for the relief of vasomotor symptoms, while oral O+P is graded lower with additional flaws and side effects. Although the effectiveness of CR extract is comparatively lesser than that of the well-established commercially available HRT, it is a safer and hormone-free remedy for postmenopausal syndrome. No research outcomes supported the estrogenicity of the extract, and hence it could be safely used in breast cancer patients.

The present review clearly encapsulates the use of CR extract for effective and safe therapy to alleviate menopausal symptoms. However, there is no culmination regarding the association of specific phytochemical constituents with pharmacological action. CR can be used successfully as a potential alternative to HRT in adult women, but demands long-term clinical safety research. Even though plenty of studies have concentrated on explaining the functionality of the extract, unfortunately, its mechanism of action is as yet ambiguous. Therefore, additional research is indispensable to fully comprehend the mechanism of action of this well-admired botanical. Additionally, in order to attain its faithful usage in a clinical setting, it is desired that formulation scientists should pay attention to the innovation of a safe, cost-effective, patient-compliant product of CR which would be helpful for many adult women for the mitigation of menopausal distress.

## Figures and Tables

**Table 1:** Available commercial products of CR on the US market.

Sl. No	Products	Serving Size	Amount (mg) of CR Extract/1 Serving Size	Amount of Triterpenes/1 Serving Size (mg)
1	Remifemin	1 tablet	-	40
2	Enzymatic Therapy Remifemin	1 tablet	-	20
3	XYMOGEN MenoFem	1.0 Capsule(s)	50	-
4	Vitanica Women’s Passage	1.0 Capsule(s)	40	-
5	Estroven Extra Strength Estroven	2.0 Caplet(s)	40	-
6	Irwin Naturals EstroPause	4.0 Liquid Softgel(s)	80	-
7	Lydia E. 8Pinkham Herbal Liquid Supplement with Vitamins C and E	1.0 Tablespoon(s)	-	-
8	Gaia Herbs Single Herbs Black Cohosh	1.0 Capsule(s)	400	2
9	Oregon’s Wild Harvest Black Cohosh	1.0 Capsule(s)	300	-
10	Natrol Black Cohosh Extract 80 mg	2.0 Capsule(s))	160	4
11	Major Black Cohosh 40 mg	1.0 Capsule(s)	-	40
12	Bluebonnet Standardized Black Cohosh Root Extract	1.0 Capsule(s)	250	6.25
13	Gaia Herbs SystemSupport Women’s Balance	1.0 Capsule(s)	200	1
14	Nature’s Way Black Cohosh Root	3.0 Capsule(s)	540	-
15	Oregon’s Wild Harvest Black Cohosh	1.0 mL	500	-
16	NOW Red Clover/Black Cohosh 225 mg/40 mg	Serving size: 1.0 Vcap(s)(R)	40	-
17	Vitamin World Extra Strength Black Cohosh 40 mg	1.0 Softgel(s)	40	1
18	Terry Naturally Menopause Relief plus	2 capsule(s)	-	-
19	Botanic Choice Black Cohosh Root	1.0 mL	35	-

**Table 2:** Different chromatographic techniques for the evaluation of CR triterpenes.

Title of the Manuscript	System and Detector	Plate or Column Used	Solvent System/Mobile Phase	Advantage	Reference
“Actaea racemosa (root and rhizome)”	HPTLC-anisaldehyde reagent	TLC plate having coating of Silica gel 60, F254	Toluen:ethyl formate:formic acid (5:3:2, *v*/*v*/*v*)	Simple method	[35]
“Fast analysis of Triterpene glycosides in CR using Agilent 1290 Infinity LC system and Agilent Poroshell 120 SB c-18 2.7 µm”	1290 infinity LC system ELSD	Poroshell 120 SB C-18 2.7 µm column. Agilent 385 ELSD, Model-G4 261 A.	A Solvent: 1% formic acid in water B Solvent: Acetonitrile	This is a revised method for the established USP method of analysis of CR triterpenes. A more efficient approach than USP condition in terms of time and solvent consumption. Here, all the conditions (mobile phase, detector setting and column temp) are the same except for column	[36]
“Determination of Triterpene Glycosides in Cimicifuga racemosa (Black Cohosh) by HPLC-CAD”	HPLC-CAD	C18 HPLC column, 4.6 × 150 mm, 2.7 μm particle size	A Solvent: 0.1% formic acid in water B Solvent: Acetonitrile	Here, the calibration curves and signal-to-noise ratios for ELSD and charged aerosol detection of the triterpene glycoside (27-deoxyactein) in a black cohosh extract are compared. It can be concluded that the Thermo Scientific Dionex Corona™ CAD™ Charged Aerosol detector has ↑ sensitivity, calibration linearity, and reproducibility over ELSD	[37]
“Detection of Actaea racemosa Adulteration by Thin-Layer Chromatography and Combined Thin-Layer Chromatography-Bioluminescence”	HPTLC and HPTLC Bioluminescence (For identification of adulterants)—5% H_2_SO_4_–anisaldehyde reagent, Vibrio fischeri culture	Bioluminex silica gel 60 F254 HPTLC plates	Toluene:ethyl formate:formic acid (5 + 3 + 2, *v*/*v*/*v*).	An efficient, economical, and effective technique that helps to identify common adulterants in black cohosh. Unknown contaminants that were not identified by standard identification techniques were easily identified by this	[38]
“The HPLC Analysis of CR Using an INA Method”	HPLC-ELSD	Phenomenex Prodigy ODS-3, 5 μm, 250 × 4.6 mm	A Solvent:0.1% formic acid B Solvent: Acetonitrile	---	[39]
“Isolation, structure elucidation, and absolute configuration of 26-deoxyactein from Cimicifuga racemosa and clarification of nomenclature associated with 27-deoxyactein”	HPLC-ELSD	YMC ODS-AQ RP-18 column (5 μm, 120 Å, 4.6 × 250 mm)	Solvent A: water containing 0.05% TFA, Solvent B: acetonitrile, Solvent C: water	Helps to isolate 3 triterpenes from the roots/rhizomes of CR	[40]
“Stability Evaluation of Selected Polyphenols and Triterpene Glycosides in CR”	HPLC-PDA	125 × 4.0 mm i.d. Hypersil ODS column-quantitative 250 × 4.6 mm i.d., 5 μm Aqua C18 column-qualitative	A Solvent: 5% (*v*/*v*) acetonitrile B Solvent: Water	Evaluates the stability of the triterpene glycosides present in CR (plant material, extracts, and encapsulated commercial extract). With an HPLC-PDA method, 3 triterpene glycosides in CR were quantitatively measured for a specific period and were found stable at the tested conditions	[41]
“Quantitative determination of triterpenoids and formononetin in rhizomes of black cohosh (Actaea racemosa) and dietary supplements by using UPLC-UV/ELS detection and identification by UPLC-MS”	UPLC-UV/ELS	UPLC BEH C18 column (100 mm × 2.1 mm i.d., 1.7 μm	Gradient elution with water and acetonitrile: methanol (7:3) at a constant flow rate of 0.3 mL/min n, 55% A/45% B; in the next 7 min, 35% A/65% B using a slightly concave gradient profile	Successfully used to examine the various commercial product of CR along with differentiating between 2 other Actaea species. It has been concluded that there was significant inconsistency in the quantities of the selected triterpenes for different products of black cohosh	[33]
“Direct analysis and identification of triterpene glycosides by LC/MS in black cohosh, Cimicifuga racemosa, and in several commercially available black cohosh products”	HPLC-PDA	Hypersil ODS column-(5 μm, 4ID × 125 mm	A Solvent: water B Solvent: acetonitrile	Used to differentiate CR products from different plant species for quality control reasons	[42]
“HPLC Analysis of Triterpene Glycosides in Black Cohosh Formulations using the PL-ELS 2100”	PL-ELS 2100 (neb = 30 °C, evap = 50 °C, gas = 1.4 SLM) UV-Vis @ 230 nm	Inertsil C18 5 µm, 150 × 4.6 mm	A Solvent: 0.1% Formic Acid in Water B Solvent: Acetonitrile	To certify the potency of commercially available black cohosh tablets	[43]
“Phytochemical Fingerprinting to Thwart Black Cohosh Adulteration: a 15 Actaea Species Analysis”	HPLC-PDA-LCMS	Triterpene glycosides were performed with a 125 × 4.0 mm i.d. Hypersil ODS column (Agilent, Santa Clara, CA, USA	Step gradient starting with 5% (*v*/*v*) acetonitrile (A) in water (B) and ↑ to 100% acetonitrile over 60 min	A practical, reliable method for authenticating black cohosh and differentiating it from adulterants	[44]
“Evaluation of the Botanical Authenticity and Phytochemical Profile of Black Cohosh Products by High-Performance Liquid Chromatography with Selected Ion Monitoring Liquid Chromatography−Mass Spectrometry”	HPLC-PDA -LCMS (APCI)	150 mm × 3.9 mm i.d., 5 µm, Waters C18 column	Step gradient starting with 5% (*v*/*v*) acetonitrile (solvent A) in water (Solvent B)	11 black cohosh products were analyzed for triterpene glycosides and other constituents by using HPLC-PDA and a newly selected ion monitoring LC-MS method. The study concluded that the product contained Asian Actaea as a replacement for black cohosh	[45]
“Chemical profiling of Actaea species and commercial products using UPLC-QTof-MS”	UPLC-QTof-MS	Acquity UPLC HSS T3 2.1 × 100 mm,1.8 μm 40 °C	Gradient elution with Solvent A: 0.1% formic acid in water Solvent B: acetonitrile	Helps to recognize useful marker compounds such as cimifugin derivatives, triterpenes, and alkaloids that distinguish between Actaea species based on exact mass precursor ion, theoretical isotopic distribution, and high-energy fragment ion data	[46]
“The value of plant collections in ethnopharmacology: a case study of 85-year-old black cohosh (Actaea racemosa L.) sample”	HPLC- PDA- LCMS	C18 column (3.9 mm × 150 mm, 5 m)	Solvent A: water Solvent B: acetonitrile	A comparative study to confirm stability between the ingredients of the 85-year-old plant sample with that of a new collection of *Actaea racemosa* by quantitative study. Both plant samples have comparable quantities of the 4 major triterpene glycosides, thus concluding the similarity of both samples and confirming the stability of the older sample	[47]
“Species Identification of Black Cohosh by LC-MS for Quality Control”	Reversed-phase liquid chromatography with positive atmospheric pressure chemical ionization mass spectrometry (LC/(+)APCIMS			A fast and accurate method for analyzing the 4 triterpene: actein, 27-deoxyactein, cimicifugoside M and cimicifugoside from CR for quality control purposes	[48]

**Table 3:** Pharmacologically active constituents of CR.

Source	Compound Class and Name	Part of the Plant	Reference
*Cimicifuga racemosa*	Cimigenol-3-O-β-D-xyloside (Cimigenoside)	Rhizome	[59]
	25-O-Acetylcimigenol-3-O-β-Dxyloside (25-O-Acetylcimigenol xyloside	Rhizome	[60]
	Cimiracemoside A	Rhizome	[44,61]
	(24S)-24-O-Acetylhydroshengmanol- 3-O-β-D-xyl-Δ16,17-enol ether	Root and rhizome	[62]
	23-epi-26-Deoxyactei	Root and rhizome	[63]
	26-Deoxyactein (=27-Deoxyactein)	Root and rhizome	[40]
	Actein	Root and rhizome	[40,63]
	Caffeic acid	Root and rhizome	[64]
	Cimiracemate A	Rhizome	[60]
	Cimiracemate B	Rhizome	[65]

**Table 4:** Chemical structures of important constituents present in CR.

Name of the Constituent	Chemical Structure
Cimiracemate A	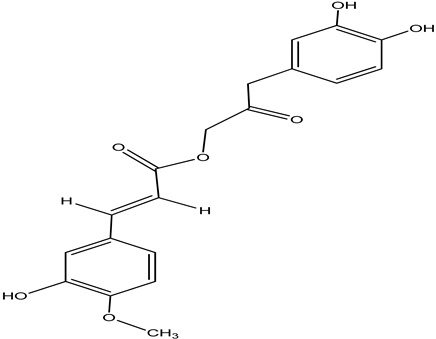
Cimiracemate B	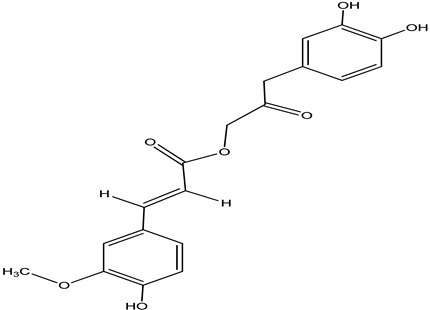
Caffeic acid	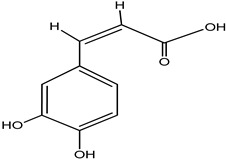
n-methyl serotonin	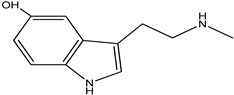
isoferulic acid	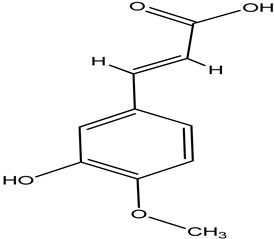
Actein	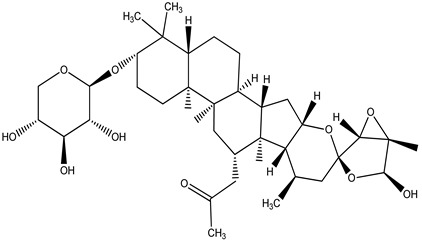
26-Deoxyactein	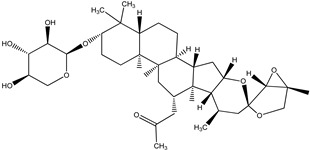
23-epi-26-Deoxyactei	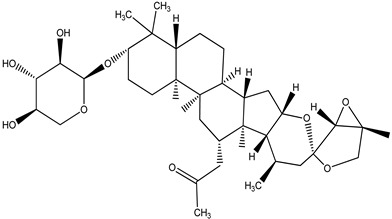
Cimigenol-3-O-β-D-xyloside (Cimigenoside)	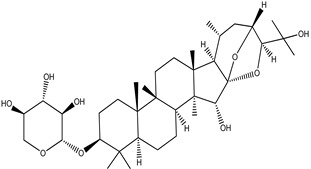
25-O-Acetylcimigenol-3-O-β-Dxyloside (25-O-Acetylcimigenol xyloside	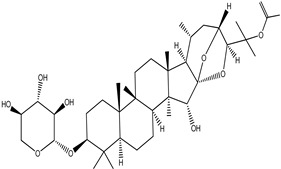
25-O-Anhydrocimigenol-3-O-β-Dxyloside (25-Anhydrocimigenol xyloside	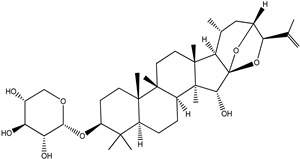
Cimiracemoside A	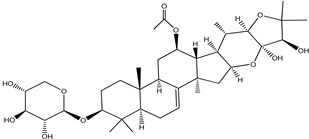
Cimiracemoside B	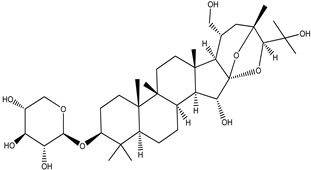
Cimiracemoside C	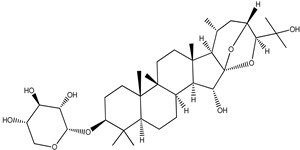
Cimiracemoside D	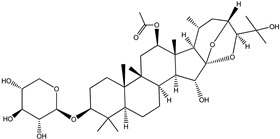
Cimiracemoside E	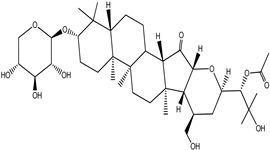
Cimiracemoside F	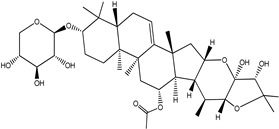
Cimiracemoside G	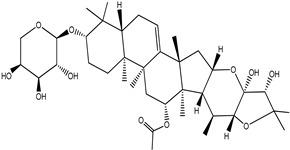
Cimiracemoside H	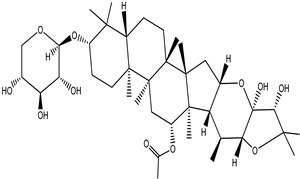
Cimiracemoside M	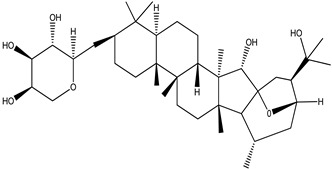

**Table 5:** Different quality control methods available for CR.

Sl. No.	Purpose	Analytical Method Used	Conclusion	Reference
1	A quality control method of CR products used to differentiate CR products from among other plant species	Employed reversed-phase LC-MS to identifies triterpene glycosides	Identified several triterpenes, such as 27-deoxyactein, actein, cimicfugoside M, and cimicifugoside, from CR; also identified a chromone, cimifugin, from *C. foetida*. Study can conclude that Cimicifugoside M and cimifugin can act precisely as a marker for the identification of species	[42]
2	Used to distinguish species of Cimicifuga and other botanicals for women’s health issues. This methodology can be used to recognize species of commercial value without considering collection time or geographic area	Employed random amplified polymorphic DNA (RAPD) analysis	The study identified species-specific DNA fragments and concluded that Cimicifuga species collected from diverse geographical locations have the same profiles but not matched DNA	[66]
3	Analysis of the methanolic extract of CR root and its methanolysis products	Employed GC-MS method	Identified the marker that is most specific for the identification of this plant: 2-Hexylcyclopropaneoctanoic acid (9,10-methylenehexadecanoic acid). Moreover, developed a validated method for the quantitative analysis of different compounds such as isoferulic acid, formononetin, and total triterpene glycosides in CR root	[54]
4	This method is used to observe the chromatography or fingerprint profile of 7 CR herbs and 6 CR commercial samples	LC/turbo ion spray (TIS)- MS method	Provides a reliable and reproducible method that can be used for botanical identification of CR plants and the inspection and validation of CR commercial samples. Different chromatograms patterns were developed for different species of CR, such as *Cimicifuga racemosa*, *Cimicifuga dahurica*, *Cimicifuga foetida*, *Cimicifuga heracleifolia*, *Cimicifuga japonica*, *Cimicifuga acerina*, and *Cimicifuga simplex*, by using LC/MS. 23-Epi-26-deoxyactein was present only in CR, *C. dahurica*, and C. foetida and were quantified by LC/MS/MS method	[68]
5	Method used for the botanical standardization and quality control of CR products	HPLC-PDA/MS/ELSD	Developed and validated using 10 Cimicifuga species. The triterpenes cimifugin, cimigenol-3-O-arabinoside, and cimifugin-3-O-glucoside were identified as appropriate species-specific markers for the identification of CR from the other species of Cimicifuga. It offers the identification as well as perception into chemical inter conversion methods occurring among the various triterpenes in CR	[34]
6	This is used for the identification of CR and recognition of its common adulterants by fingerprint profiles	HPTLC method	A practical, fast, and reliable method with specific derivatization reagents. This allows the detection of mixtures of CR with a minimum of 5% of the adulterants. Can be used for quality control of CR raw material in a current good manufacturing practices environment	[69]
7	Phytochemicals method was developed to differentiate four different groups of Actaea	HPLC-TOF-ESI-MS technique and principal component analysis	Used for metabolic profiling to distinguish CR from related species of Actaea. It identified 15 chemical markers where 3 markers were recognized using reliable standards, and 12 marker compounds were tentatively recognized by comparing the fragmentation patterns with past information. The occurrence of these marker compounds is critical for the identification of 4 groups of closely associated plants	[70]
8	This method is used for polyphenols and triterpene glycosides	HPLC and LC-MS fingerprints	Showed different patterns that make CR distinguishable from several other Actaea species. Cimifugin and cimiracemoside F are the two marker compounds, which were found to be essential to differentiate CR from most Asian species of Actaea	[44]
9	Identified 2 matK nucleotides that constantly discriminate CR from other correlated species and correctly identified the CR samples	Employed nucleotides	Out of 36 dietary supplements sequenced, 75% had a sequence that accurately paired with CR and the remaining 25% had a sequence identical to that of 3 Asian Actaea species (*A. cimicifuga, A. dahurica, and A. simplex*)	[71]
10	Proposed 2 metabolic fingerprinting methods to identify and validate Actaea species	DNA sequencing in combination with flow injection MS and proton NMR spectrometry	Single-source CR samples were differentiated from other species based on key component analysis, soft independent modelling of class analogies of flow injection MS and proton NMR spectrometry metabolic fingerprints. DNA sequence information from 2 independent gene regions confirmed the metabolic fingerprinting by DNA sequencing. Moreover, the combined CR samples were distinguishable from commercial root samples and commercial products	[72]
11	Tried to establish an analytical method to evaluate the authentication of the plant materials and finished products	Polymerase chain reaction-restriction fragment length polymorphism method and a multiplex amplification refractory mutation system	Described a genome-based confirmation method and LCMS-based authentication for CR products	[73]

**Table 6:** Clinical trials of CR relating to women’s health.

Year	No. of Subjects	Study Length	Extract/Formulation/Dosage Form	Study Design	Status	Study Outcome	Reference
2002	152 Perimenopausal and postmenopausal females	6 months	2 different doses (39 mg and 127.3 mg) of CR preparation	A controlled, randomized, double-blinded parallel group study	Completed	CR extract helps in ↓ the menopause symptoms without showing estrogen-like effects and also supports the 40 mg/day standard dose of the isopropanolic aqueous CR extract over the higher dose	[95]
2003	62 postmenopausal women	3 months	With CR BNO 1055 (Klimadynon/Menofem): daily dose corresponding to 40 mg herbal drug, 0.6 mg conjugated estrogens, or matching placebo	A double-blind, randomized, multi-centre study	Completed	The results of the study concerning climacteric complaints and on bone metabolism indicate an equipotent effect of CR BNO 1055 in comparison to 0.6 mg CE/day. It is expected that CR BNO 1055 has ingredients with SERM activity	[12]
2005	64 postmenopausal women	3 Months	Either isopropanolic aqueous CR extract (40 mg daily) or transdermal estradiol (25 μ every 7 days) + dihydrogesterone (10 mg/day) for the last 12 days of the 3-month estradiol treatment	A randomized clinical study	Completed	CR (40 mg/day) may be a valid substitute to low-dose transdermal estradiol in managing the climacteric complaints of women who cannot be treated with conventional approaches	[80]
2005	122 menopausal women	12 weeks	CR extract	A multi-center, randomized, placebo-controlled, double-blind, parallel-group study	Completed	CR extract is superior in comparison to placebo in patients having menopausal syndromes of modest strength	[96]
2006	351 women	12 months	Black cohosh (160 mg daily); multi-botanical having black cohosh, (200 mg daily) with 9 other ingredients	A randomized, double-blind, placebo-controlled trial	Completed	Black cohosh used singly, or as a component of a multi-botanical treatment, reveals little potential as a vital remedy for vasomotor problems	[97]
2007	74 women	6 months	Black cohosh (40 mg daily)	A prospective, open, uncontrolled drug safety study	Completed	There are no harmful effects of the isopropanolic extract of black cohosh on breast tissue and also no indication of any endometrial or general safety concerns in the course of treatment	[98]
2009	88 women	12 months	Black cohosh (128 mg/day)	A randomized, four-arm, double-blind clinical trial	Completed	Black cohosh and red clover did not ↓ the number of vasomotor signs compared with placebo but standardized extracts of black cohosh and red clover were safe biologically and chemically during daily administration for the total period	[99]
2010	128 women	10 weeks	Black cohosh (40 mg/day)	A randomized, controlled trial	Completed	Adjuvant supplementation of black cohosh did not ↑ our exercise schedule, positively affect bone, menopausal symptoms, lean body mass, or to a lesser degree, 10-year CHD risk in early postmenopausal females	[100]
2011	65 women	6 months	Black cohosh	A prospective, double-blind, placebo-controlled study	Completed	Black cohosh does not affect mammographic breast density	[101]
2011	50 Tamoxifen treated breast cancer patient	6 months	Isopropanolic extract of black cohosh (1–4 tablets, 2.5 mg)	A prospective observational study	Completed	Breast cancer patients treated with tamoxifen, mainly having psychovegetative indications can be reasonably treated with black cohosh extract	[102]
2012	304 women		Standardized isopropanolic extract of black cohosh (Remifemin)	A randomized, double-blind, placebo-controlled, multicenter clinical study	Completed	Remifemin is efficacious and tolerable in the management of menopausal symptoms, specifically hot flashes	[103]
2013	84 women	8 weeks	Dried extract of Black cohosh roots (6.5 mg daily orally).	A randomized, double-blind, placebo-controlled clinical trial	Completed	Black cohosh reduced the Greene climacteric scale (GCS) total score, and all GCS subscale scores (vasomotor, psychiatric, physical, and sexual symptoms) during the period of treatment.	[104]
2013	120 women	2 months	Remifemin (one tablet twice daily) and Paroxetine (20 mg once daily)	A randomized, controlled trial	Completed	The combined treatment of Remifemin and Paroxetine can ↑ the efficacy of perimenopausal depression and also has a ↑ safety profile with lesser side effects	[105]
2014	116 women	12 weeks	Remifemin (20 mg) and Tibolone (2.5 mg)	A randomized study	Completed	Remifemin had a parallel clinical efficacy as compared to Tibolone and was safer for the peri-menopausal symptoms induced by GnRH-a in endometriosis patients	[106]
2015	48 women	6 months	Black cohosh extract (Oral)	A randomized, double-blind and placebo-controlled research	Completed	Black cohosh extract effectively ↑ sleep in early postmenopausal females with major sleep complaints and might be a safe measure in managing menopausal sleep disturbance	[107]
2015	54 women		Black cohosh extract (40 mg/day)	A randomized, double-blind, placebo-controlled clinical trial	Completed	Black cohosh extract was not more efficient than placebo for alleviating moderate to serious menopausal symptoms or ↑ quality-of-life scores in Thai women	[108]
2019	85 women	12 weeks	Remifemin, the commercialized product of CR extract, combined with LHRH-a.	A perspective randomized-design study	Completed	CR is efficient, reliable and safe for the management of menopausal syndrome caused by luteinizing-hormone releasing hormone analogue in breast malignancy	[109]
2020	174 women	12 months	CR extract, Ze 450 and menopausal hormone therapy	A monocentric retrospective cohort study	Completed	Menopausal symptoms ↑ significantly in both groups (MHT and CR), without altering the serum metabolic parameters and body weight	[110]

**Table 7:** Patents of CR relating to women’s health.

Patent No.	Title	Disease/Problem	Proof of Concept
US20040202736A1 United States 2004	Method of ameliorating side effects of SERMs	Side effects of selective estrogen receptor modulators (SERMs).	The present invention narrates a method of treatment and/or prevention of side effects such as hot flashes caused by SERMs such as tamoxifen, by administering an effective amount of a standardized dry extract of CR over a particular period
US6713097B2 United States	Use of preparation of Cimicifuga racemosa	Females having urinary incontinence following an ovariohysterectomy/hysterectomy/menopause	The present invention describes a specific preparation from the rhizome of CR which can be used for the successful treatment of urinary incontinence in females following an ovariohysterectomy. Positive results can also be expected for females having a hysterectomy or after menopause
WO1998026791A1 WIPO (PCT)	The use of a CR extract	Estrogen-dependent tumors	This innovation relates to the use of an extract of CR for the treatment of estrogen-dependent tumors. This invention describes that the simultaneous administration of a CR extract (doses of 5 to 500 mg/day) with an anti-estrogenic active substance will enhance its action on estrogen-dependent tumors
US20120071501A1 United States 2012	Use of extracts of the genus cimicifuga as organo selective medicines for treating diseases of the genitourinary tract caused by sex hormones.	Genitourinary tract infection is caused by sex hormones.	Describes CR extracts suitable for making a ready-formulated drug for the selective treatment and/or prevention of sexual hormone-related ailments of the urogenital tract, post-menopausal urinary bladder infections and for the treatment of benign and malignant prostate hyperplasia
EP2545932A1 European Patent Office 2013	Selected cimicifuga fractions for the treatment of osteoporosis	Osteoporosis	The invention relates to the methods for producing cimicifuga fractions from a Cimicifug extract, for the prevention and treatment of osteoporosis in humans and animals
US20060210659A1 United States	Anti-obesity agent	Obesity	The present invention provides the use of a CR plant in order to prepare an active substance which has an anti-obesity effect such as a blood triglyceride-lowering agent, a cholesterol-lowering agent, a body fat storage suppressive agent, an anti-obesity agent and an anti-lipemic agent comprises a cycloartane-type triterpene or glycoside thereof. Additionally, the present invention provides a beverage, food, and quasi-drug comprising of said agents
WO2020144588A1 WIPO (PCT)	Compositions for treatment of menopause, osteopenia, and osteoporosis, and menopause/related metabolic and vascular disorders.	Menopause, osteopenia and osteoporosis, and menopause/related metabolic and vascular disorders	The present invention discloses a composition comprising of CR and Ferula extracts, and optionally other phytotherapeutic extracts, vitamins, and oligo-elements for the treatment of symptoms related to menopause
US20030224068A1 United States 2003	Compounds for hormonal therapy	Hormonal therapy	The present invention offers a therapeutic composition consisting of a therapeutically effective amount of 27-deoxyactein. It also describes a multi-step process for the isolation of 27-deoxyactein from CR by chromatographic suitable techniques

**Table 8:** Viewpoints of different regulatory agencies/professional bodies regarding liver toxicity relating to CR.

Regulatory Agencies/Organizations	Recommendations/Conclusions	Reference
Australian Department of Health (Therapeutic Goods Administration) 2007	They reviewed the existing regulatory controls on CR and concluded that there is a link between the use of CR and liver injury; however, it is very unusual. They determined that although CR shows rare liver-damaging properties, it is still suitable for use in complementary medicines, with proper warning statements such as: “Warning: CR may harm the liver in some individuals. Use under the supervision of a healthcare professional” on the product label	[129]
Health Canada	Health Canada suggests that consumers using CR products should use them with caution and if they have any concerns regarding its use they should refer to a physician. If the consumers have a weakness, loss of appetite, rare fatigue, or if they develop signs indicative of liver damages such as in the whites of the eyes or the yellowing of the skin, abdominal pain or dark urine, they should immediately terminate the use of the product and refer to a physician	[130]
Medicine and Healthcare products Regulatory Agency (MHRA), UK	MHRA suggests that warnings must be included in the product info for CR, relating to the rare harmful reactions in the liver for both registered and unregistered goods. The liver toxicity concern of the CR should be observed carefully, and further evidence should be collected on the quality of CR products and their composition accessible in the UK market. Furthermore, the potential mechanism of CR products associated with liver injury should be studied	[131]
The U.S. Pharmacopeia, 2008	USP recommends CR products must be labelled with a cautionary statement such as: “Discontinue use and consult a healthcare practitioner if you have a liver disorder or develop symptoms of liver trouble, such as abdominal pain, dark urine, or jaundice”. Nevertheless, the U.S. FDA does not need such caution on labels of CR products	[125]
The American Herbal Products Association, 2013	They recommend that CR should be avoided in pregnant women apart from females under the care of their healthcare professional	[8]

## Data Availability

Data sharing not applicable.
